# A Road Less Traveled: E-test Method for Antifungal Susceptibility Testing in Trichophyton mentagrophyte Isolates Among Patients Presenting With Dermatophytosis at a Tertiary Healthcare Center in North India

**DOI:** 10.7759/cureus.62047

**Published:** 2024-06-10

**Authors:** Kriti Maurya, Nikhil Raj, Amit Kumar Singh, Anupam Das, Manodeep Sen, Jaya Garg, Jyotsna Agarwal

**Affiliations:** 1 Microbiology, Autonomous State Medical College, Hardoi, IND; 2 Microbiology, Dr. Ram Manohar Lohia Institute of Medical Sciences, Lucknow, IND; 3 Microbiology, Prasad Institute of Medical Sciences, Lucknow, IND

**Keywords:** ringworm, e-test, afst, trichophyton mentagrophyte, dermatophytosis

## Abstract

Introduction

Dermatophytosis is a common infection of the skin, hair, and nails caused by dermatophytes, a group of filamentous fungi capable of digesting and obtaining nutrients from keratin. Dermatophytes comprise three important genera: *Epidermophyton*,* Microsporum*,and *Trichophyton. *This study aimed to analyze the antifungal susceptibility patterns of *Trichophyton mentagrophytes* isolates using the epsilometer test (E-test) method.

Material and methods

This prospective observational study was conducted on clinically suspected cases of dermatophytosis. All samples, including skin scrapings, hair, and nails, were subjected to potassium hydroxide (KOH) examination followed by fungal culture. The *Trichophyton mentagrophytes* isolates were then subjected to antifungal susceptibility testing using the E-test method for the two most prescribed antifungals: itraconazole and fluconazole.

Results

In this study, one-third of the patients who tested positive for dermatophytosis belonged to the same family, with spouses being the most commonly affected. Tinea corporis was the most common clinical presentation, with *Trichophyton mentagrophytes *identified as the most common etiological agent. Itraconazole was more effective than fluconazole.

Conclusion

The current study demonstrated that antifungal susceptibility testing of dermatophytes using the E-test is easier and can be applied in routine laboratories as a screening method, serving as an alternative to broth microdilution.

## Introduction

Dermatophytosis, a superficial fungal infection, is a very common problem worldwide [1]. It is caused by dermatophytes, which include three important genera of filamentous fungi:* Epidermophyton*,* Microsporum*, and *Trichophyton* [2]. Its clinical appearance is frequently mistaken for that of other skin conditions, primarily because of the widespread use of steroid-based ointments and inappropriate antifungals, resulting in incorrect diagnosis and inappropriate management [3]. Additionally, there has been a shift in the recurrence rate, treatment response, severity, and presentation of the disease.

Approximately 20-25% of the population is affected by superficial fungal infections globally [4]. Crowded living conditions, poverty, poor hygiene, outdoor labor, increased physical exertion, and excessive perspiration all increase the likelihood of developing dermatophytosis [5]. Studies conducted in recent years have revealed a rising trend in the prevalence, as well as a shift in the spectrum of infection and the isolation of previously uncommon species. Previous research from India identified *Trichophyton rubrum* as the most prevalent agent causing dermatophytosis; however, *Trichophyton mentagrophyte* has emerged as the primary causal organism, with strong terbinafine and azole resistance, according to recent studies [6,7].

Dermatophyte infections can be reliably diagnosed based on patient history and clinical examination; however, a clinical diagnosis needs to be supported by laboratory diagnosis [8]. The diagnosis of dermatophyte infection should be confirmed by potassium hydroxide (KOH) mount microscopy, culture, or histopathological examination. Culture is essential to support direct microscopic examination for identifying the causative agent [9]. In numerous cases, selecting the appropriate treatment is possible only by accurately identifying an invasive mold, which is crucial for treating infections caused by non-dermatophyte filamentous fungi, as these fungi often resist the standard therapy dosages used for treating dermatophyte infections [10].

There is very limited published research and literature from Uttar Pradesh (North India) describing the drug sensitivity patterns and clinico-mycological features of dermatophytosis. Additionally, there are limited studies on the antifungal susceptibility of dermatophytes using the epsilometer test (E-test) method. With this background, this study investigated the clinico-mycological correlation in patients infected with dermatophytes and the in vitro activity of two commonly used antifungal agents (itraconazole and fluconazole) against *Trichophyton mentagrophyte *using the E-test method.

## Materials and methods

This was a prospective cross-sectional study conducted at the Department of Microbiology, Dr. Ram Manohar Lohia Institute of Medical Sciences (RMLIMS), Lucknow, from July 2018 to July 2019. The study focused on patients who attended the dermatology OPD at Dr. Ram Manohar Lohia Combined Hospital, Lucknow. Ethical approval was obtained from the Institute Ethics Committee (IEC), RMLIMS, Lucknow (IEC No. 32/17) before commencing the study. Patients with suspected cases of dermatophytosis presenting to the OPD were included, while patients who had received systemic or topical antifungal treatment within the last month were excluded. A total of 200 clinically suspected patients with dermatophytosis were enrolled in this one-year study.

Skin scraping was performed from the erythematous, peripheral, actively growing margins of the lesions after decontamination of the skin with 70% alcohol to remove surface bacterial contaminants. The scrapings were collected on sterile black filter paper. For nail scraping and clipping, the nails were first cleaned with 70% alcohol. Using a scalpel, the deeper portion of the nail was scraped off and collected. For scalp and hair samples, dull, lustreless hair and stubs of hair were chosen and plucked with sterile forceps.

**Figure 1:**
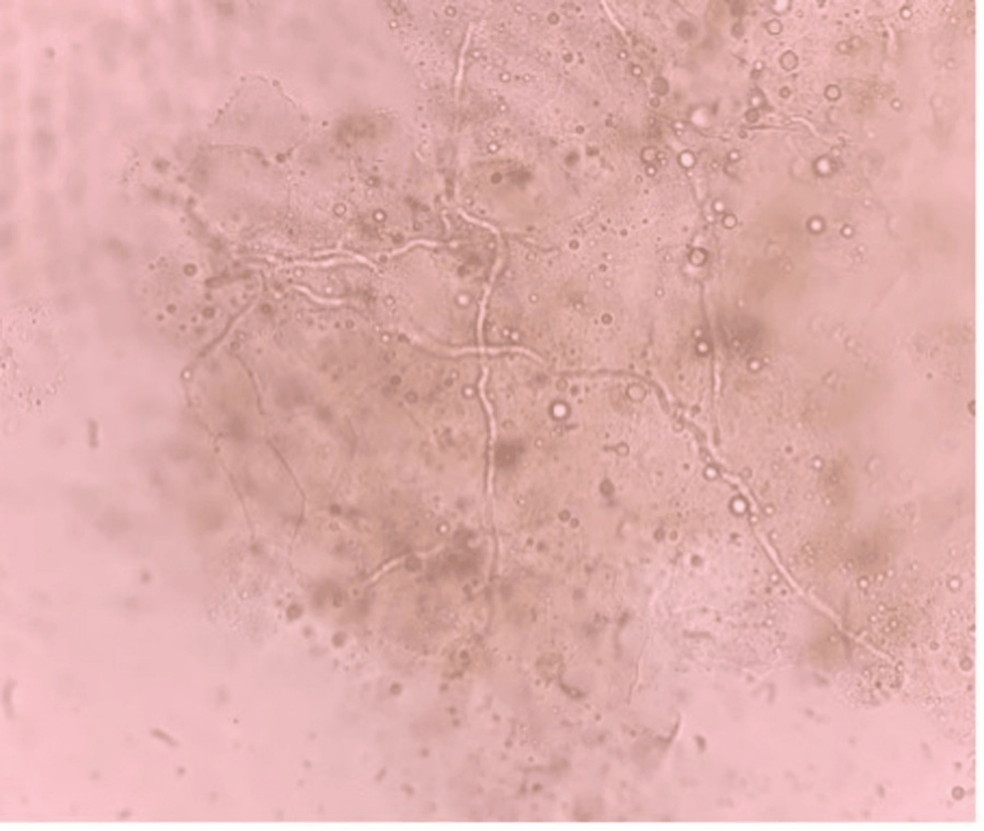
Thin septate hyaline hyphae seen on potassium hydroxide (KOH) mount under high power magnification

All the samples were transferred to a sterile glass test tube, and five to six drops of 10% KOH for skin and 20% for nails were added. After one hour, one drop of this mixture was placed on a clean, grease-free slide to observe the presence of fungal elements under low power (10x magnification) and high power (40x magnification) of the microscope (Figure 1).

**Figure 2:**
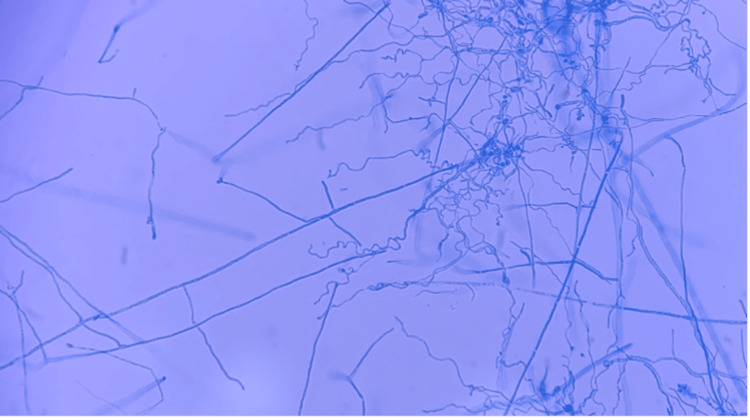
Lactophenol cotton blue mount of Trichophyton mentagrophyte under low power magnification

The samples were then cultured on Sabouraud dextrose agar (SDA) (HiMedia, Mumbai, India) and dermatophyte test medium (DTM) (HiMedia, Mumbai, India) tubes in duplicate. The samples were incubated at 25°C in an incubator for a minimum of 21 days. Colonies on the slants were examined for morphology, texture, and pigmentation (obverse and reverse) for up to 21 days. The causative fungi were confirmed by microscopic examination using lactophenol cotton blue mount and slide culture where necessary (Figure 2).

Antifungal susceptibility testing of Trichophyton mentagrophyte

The selected *Trichophyton mentagrophyte* isolates were subjected to antifungal susceptibility testing using E-test strips for common antifungals such as itraconazole and fluconazole. The E-test strips feature a predefined, exponential, and continuous gradient of antifungal agents across 15 two-fold dilutions. A fresh subculture on potato dextrose agar was done from the mother tubes and incubated. The resulting growth was mixed with 2 ml of 0.9% normal saline to create an inoculum corresponding to a concentration of 10^5^ to 10^6^ colony-forming units (CFU)/ml [11,12]. Roswell Park Memorial Institute (RPMI) 1640 agar (HiMedia, Mumbai, India) was inoculated by dipping a sterile swab into the inoculum.

An E-test strip was applied to each plate, which was covered with cling wrap and incubated at 28°C in an incubator. The results were read on the third day (72 hours). The minimum inhibitory concentration (MIC) was defined as the lowest drug concentration at which the border of the elliptical inhibition zone intercepted the MIC scale on the E-test strip. For quality control and as a reference for MIC values of the E-test, the American Type Culture Collection (ATCC) strain of *Trichophyton mentagrophyte* (ATCC 18748) was used [11,12].

## Results

A total of 200 patients were included in the study. Of these, 157 tested positive for fungal hyphae upon KOH mount examination, and 142 were culture-positive. Among the 142 culture-positive patients, 120 (84.5%) exhibited dermatophyte growth, while 22 (15.5%) exhibited non-dermatophyte growth.

The prevalence of dermatophytes was notably higher among males, accounting for 104 cases (86.7%) than among females, accounting for 18 cases (13.3%), resulting in a male-to-female ratio of 6.5:1. However, statistical analysis did not reveal a significant correlation between sex distribution and dermatophytosis (p=0.547). Regarding the age-wise distribution, the most affected age group was 16-30 years (Table 1).

**Table 1:** Age and gender distribution of dermatophyte cases

Age group (n)	Male gender n (%)	Female gender n (%)
<15 years (7)	6 (85.7)	1 (14.3)
16-30 years (66)	58 (87.8)	8 (12.2)
31-45 years (30)	25 (83.3)	5 (16.7)
46-60 years (12)	10 (83.3	2 (16.7)
61-75 years (5)	5 (100)	0 (0)
Total (n=120)	104 (86.7)	16 (13.3)

**Figure 3:**
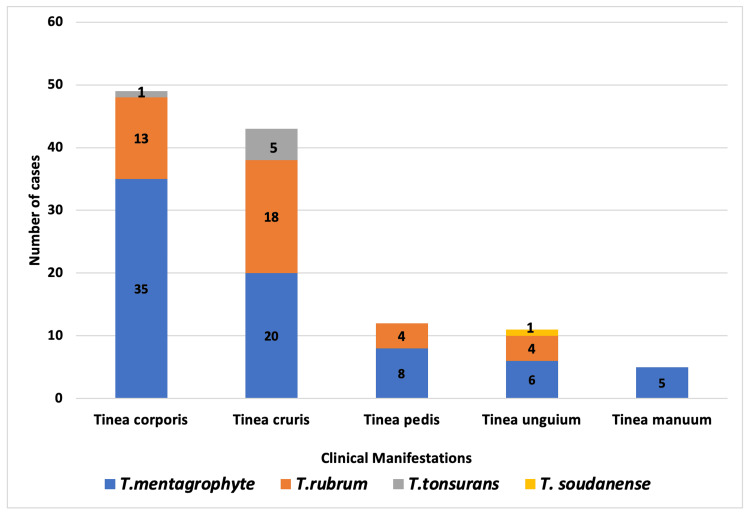
Correlation of clinical manifestations with dermatophyte isolates

Of the 120 patients with dermatophyte infection, 39 (32.5%) had a history of contact with patients suffering from fungal infections. Most patients presented with tinea corporis (40.8%), followed by tinea cruris (35.8%), tinea pedis (10%), tinea unguium (9.2%), and tinea manuum (4.2%) (Figure 3).

Among the 120 dermatophyte isolates analyzed, 74 (61.7%) were *Trichophyton mentagrophyte*, followed by *Trichophyton rubrum*, which was detected in 39 (32.5%) patients, and *Trichophyton tonsurans*, which was detected in 6 (5%) patients. Additionally, a rare isolate of *Trichophyton soudanense* was identified in a patient who had recently returned from the Philippines after a brief stay there for a few months.

Of these, 74 *Trichophyton mentagrophyte* isolates and 25 representative isolates were subjected to antifungal susceptibility testing using the E-test method along with the ATCC (18748) strain of *Trichophyton mentagrophyte*. These 25 strains were tested for the two most commonly prescribed antifungals, i.e., fluconazole and itraconazole. In this study, we found that itraconazole was more active, as the dermatophyte isolates had lower MIC values ranging from 0.002 µg/mL to 0.25 µg/mL, compared to fluconazole, which had an MIC range of 16-256 µg/mL. Itraconazole had low MIC values ranging from 0.002-0.094 µg/mL in 19 (76%) isolates. On the other hand, fluconazole was not very effective in vitro; 19 (76%) isolates were fully resistant to fluconazole, and the MIC was >256 µg/mL. The MIC of itraconazole for the ATCC strain was 0.125 µg/mL, and that for fluconazole was 64 µg/mL.

## Discussion

In the present study, among all culture-positive patients, 120 (84.5%) cases had dermatophyte infection, and 22 (15.5%) cases had non-dermatophyte infection. Our findings were in agreement with those of Lakshmanan et al., who reported that 75.6% of all superficial fungal infections were dermatophytosis cases, while 24.4% were non-dermatophytosis cases [13]. Out of 120 patients whose samples grew dermatophyte fungi, 104 (86.7%) were male, and 16 (13.3%) were female, with a male-to-female ratio of 6.5:1. Nussipov et al. observed a similar distribution and reported that 68.2% of dermatophytosis cases were male, and 31.8% were female, with a male-to-female ratio of 2.14:1 [14]. Similarly, Alemayehu et al. reported an increased incidence of dermatophytosis in male children [15]. Male predominance is seen in dermatophyte infection as they are more likely to engage in activities that expose them to environments conducive to dermatophyte growth. This includes physical labor, sports, or other outdoor activities that involve frequent sweating, and contact with contaminated surfaces.

The results of this study indicate that dermatophyte infections are more prevalent in young adults, regardless of gender. This is likely due to working environments that expose them to hot and humid conditions. Additionally, personal hygiene and the nature of their jobs contribute to the higher incidence of dermatophytosis among young adults. In this study, 40% of patients who were culture-positive for dermatophyte infection had tinea corporis; similarly, Ramaraj et al. reported tinea corporis as the most frequent clinical form [16]. In contrast, Alemayehu et al. reported tinea capitis as the predominant clinical form [15]. These findings suggest that various factors, such as lifestyle, culture, and economic level, may influence how dermatophyte infection manifests clinically in a particular community.

One important finding in this study was that *Trichophyton mentagrophytes* were the most common species isolated from the patients (61.7%). Earlier in most of the studies, the most common isolate used to be *Trichophyton rubrum* worldwide. Globally, Agarwalla et al., Hashemi et al., and Chadeganipour et al. have observed a similar trend, reporting *Trichophyton mentagrophyte* as the predominant cause of tinea [17-19]. In the present study, one rare isolate of *Trichophyton soudanense* was isolated from a patient who worked as an engineer in the Philippines before relocating to India.*Trichophyton soudanense* is an anthropophilic dermatophyte endemic to sub-Saharan Africa. Outside Africa, sporadic cases have been reported, and most of the cases involved African immigrants. Magill et al. reported two cases of *Trichophyton soudanense* infection in Baltimore, Maryland [20]. In India, Patwardhan et al. from southern India and Sahai et al. from north India reported cases of *Trichophyton soudanense* infections [21,22].

The majority of superficial infections caused by dermatophytes are commonly treated using topical antifungals. Oral antifungal therapy with newer agents, such as terbinafine, itraconazole, and fluconazole, is the preferred treatment for dermatophytosis that does not respond to topical therapies; however, the effectiveness of these drugs varies, resulting in treatment failure in 25-40% of patients, possibly due to inadequate patient compliance, poor bioavailability, and drug resistance [23]. In vitro, susceptibility testing of antifungal agents allows for the comparison of different antimycotics. This can help clarify reasons for a lack of clinical response and aid clinicians in selecting effective treatments for their patients.

Currently, no reference method has been established to test the drug susceptibility of dermatophytes. Dermatophytes are cultured on specific media which may take up to three weeks; once the dermatophyte has grown, antifungal susceptibility can be done using an E-test which is read after three days of incubation. The E-test is relatively easy to perform and less labor intensive as the preparation of the working solution, dilutions, and the stock solution is not required. The E-test is a promising method with broad applications in clinical laboratories for bacterial testing; however, there are only a few reports describing the use of this method in dermatophytes. In this study, we tested the antifungal susceptibility of *Trichophyton mentagrophyte* strains isolated from clinical specimens to fluconazole and itraconazole using the E-test method. In the present study, itraconazole was more effective, with a MIC range (µg/mL) of 0.002-0.25, than fluconazole, with a MIC range (µg/mL) of 16-256. Low activity of fluconazole was demonstrated in studies by Fernández-Torres et al. and Aktas et al. [11,12]. Higher MIC values for fluconazole might be attributed to issues, such as interaction with certain culture medium components or insolubility of the drug at higher concentrations The readily accessible supply of fluconazole at pharmacies due to the availability of over-the-counter preparations, and the general trend of its unreasonable prescribing by quacks might all be potential causes for the widespread development of fluconazole resistance.

Limitations 

The study had a few limitations. First, only the susceptibility of *Trichophyton mentagrophytes* to two antifungal agents, itraconazole and fluconazole, was evaluated. Second, the E-test was not compared with other susceptibility testing methods in terms of accuracy and reliability.

## Conclusions

The current study demonstrated that dermatophytosis is still a common problem in developing countries and that tinea corporis is the most prevalent clinical presentation. The most frequent etiologic agent identified in the present study was *Trichophyton mentagrophyte*, which supports the fact that etiologic agent variation is related to specific geographic location, socioeconomic position, and lifestyle factors of the population under study. The observations of this study indicate that itraconazole was more effective than fluconazole when tested against isolates of *Trichophyton​​​​​​​ mentagrophyte*. Moreover, we can use E-test as a preliminary test for dermatophytes, and once resistant, they can be confirmed by the broth dilution method.
